# Reference intervals for six salivary cortisol measures based on the Croatian Late Adolescence Stress Study (CLASS)

**DOI:** 10.11613/BM.2018.010902

**Published:** 2017-11-24

**Authors:** Daniela Šupe-Domić, Goran Milas, Lada Stanišić, Irena Drmić Hofman, Irena Martinović Klarić

**Affiliations:** 1 Department of Medical Laboratory Diagnostics, University Hospital Split, Split, Croatia; 2 Centre for Research on Interindividual Differences, Institute of Social Sciences “Ivo Pilar”, Zagreb, Croatia; 3 Department of Pathology, Forensic Medicine and Cytology, University Hospital Split, Split, Croatia; 4 Department of Medical Chemistry and Biochemistry, School of Medicine, University of Split, Split, Croatia; 5 Centre for Research in Social Inequalities and Sustainability, Institute for Social Research in Zagreb, Zagreb, Croatia

**Keywords:** salivary cortisol, reference intervals, late adolescence

## Abstract

**Introduction:**

The aim of this nested study is to provide the reference intervals for already published measurements of salivary cortisol from the Croatian Adolescence Stress Study (CLASS).

**Material and methods:**

A total of 969 individuals (372 males and 597 females) were included in the reference sample (age range: 18-21 years). Salivary cortisol concentrations were determined by the enzyme immunoassay (LUCIO-Medical ELISA Salivary Cortisol Kit, Nal von Minden, Germany) in the Department of Medical Laboratory Diagnostics, University Hospital Split. Nonparametric statistics were used for calculating the reference intervals (RIs) and 90% confidence intervals (90% CIs).

**Results:**

The lower limits of RIs determined by the direct method were higher in females (> 10%) than in males for the cortisol concentrations at awakening (SCC_0_), 30 to 45 after awakening (SCC_30-45_) and at bedtime (SCC_bedtime_). The upper limits of RIs for the SCC_bedtime_ were higher (> 10%) in males than in females. Females also had higher upper limits of RIs for the cortisol awakening response (CAR) and the diurnal cortisol slope (DCS) and higher lower limits of RIs for the CAR and the area under the curve with respect to ground (AUC_G_). The lower limits of RIs for the DCS were higher in males than in females.

**Conclusions:**

Obtained reference values open the arena for introducing salivary bioscience in Croatian clinical laboratory practice and provide important data for better understanding of gender differences in adaptation to stress during late adolescence.

## Introduction

Saliva has become a biological specimen of choice in stress studies and salivary cortisol has been widely recognized as a reliable biomarker of stress exposure and functioning of the hypothalamic-pituitary-adrenal (HPA) axis (*1*). In healthy people, cortisol secretion follows a strong circadian rhythm, characterized by the highest concentrations in the morning and a decline during the day (*2*). Several indexes are used to describe the diurnal cortisol rhythm: diurnal cortisol slope (DCS), area under the curve with respect to ground (AUC_G_) and cortisol awakening response (CAR). DCS is the change in cortisol concentrations from early morning to bedtime. A steeper DCS is associated with favourable health outcomes and a flatter slope with high chronic stress and conditions such as persistent fatigue, post-traumatic stress disorder, breast cancer and coronary calcification (*1*, *3*). AUC_G_ reflects the total cortisol secretion over a period of measurement and, in general, higher AUC_G_ is linked with chronic stress (*1*). CAR is the size of post-wakening surge of cortisol that occurs in the period of 30 to 45 minutes after awakening, regulated by a mechanism distinct from the rest of the diurnal cortisol rhythm (*4*). The CAR is associated with awakening and anticipating of upcoming demands for the particular day (*4*).

Disruptions of the circadian rhythm in cortisol secretion are associated with adverse health conditions and therefore establishing health-related salivary cortisol reference values is important in the routine clinical practice. Recently, reference values were published for salivary cortisol based on a multi-centre database (CIRCORT) comprising over 18,000 individuals (*5*). However, in the CIRCORT cortisol concentrations obtained by immunoassays (either dissociation-enhanced lanthanide fluorescence immunoassay or IBL chemiluminescence assay) were converted to the reference method (tandem mass spectrometry – LC-MS/MS) and reference values were reported for the wide age strata (*e.g.* 10-year intervals for groups aged 11-80 years). In Croatia, immunoassays are the recommended method for the analyses of cortisol in serum and urine (*6*). According to the guidelines of the Croatian Chamber of Medical Biochemists (CCMB), clinical laboratories should use reference intervals published by a manufacturer that are specific for the recommended method and confirmed in the Croatian population (*6*). Since salivary analyses have not yet been implemented in Croatian clinical laboratory practice, the aim of this nested study is to provide the reference intervals for already published measurements of salivary cortisol from the Croatian Adolescence Stress Study (CLASS) (*7*).

## Materials and methods

### Study design

The sample in this nested study comes from the CLASS (*7*). The CLASS was based on a probabilistic two-stage cluster sample, stratified according to the type of public school and the city where the school was located to obtain a representative sample of students from the final grades of secondary schools from the four largest Croatian cities.

The total CLASS sample comprised of 1830 students from 26 secondary schools. Out of 1830 students, 1095 collected and returned at least one saliva sample. In accordance with the guidelines in salivary cortisol research, the following exclusion criteria were applied: use of oral contraceptives and medications containing corticosteroids, presence of endocrine disorder or recent illness and documented incompliance with the saliva collection protocol (*1*). In line with the EP28-A3C recommendations, the outliers were retained in this study (*8*). The resulting 969 individuals (372 males and 597 females) who provided useable cortisol salivary samples were included in the reference sample (median 19 years, age range: 18-21 years). All study participants signed the informed consent form.

### Saliva collection and analysis

Saliva samples were collected in accordance with the protocol for non-stimulated and passive drool (*7*). On day 1 at school, students received saliva-collecting packs and sampling instructions. On day 2, students collected saliva samples in their homes, at awakening (SCC_0_), 30 to 45 minutes post awakening (SCC_30-45 min_) and immediately before bedtime (SCC_bedtime_). Saliva samples were kept in domestic refrigerators overnight. On day 3, students brought samples to school. Collected samples were transported in portable refrigerators from school to the collection point in each city. The samples were kept at - 20 °C until sent by courier on dry ice for analyses to the Department of Medical Laboratory Diagnostics, University Hospital Centre Split.

Manufacturer’s recommendations (Nal von Minden, Germany) were followed for the analyses of salivary samples, calibration and quality control. Concentrations of cortisol were determined by using the enzyme immunoassay (LUCIO-Medical ELISA Salivary Cortisol Kit, Nal von Minden, Germany). Each analytical run included seven standard samples (0 - 220.69 nmol/L), two control samples (low: 9.05-16.77 nmol/L, mean concentration 12.91 nmol/L, and high: 93.79 - 174.34 nmol/L, mean concentration 134.07 nmol/L) and saliva samples in duplicate. According to the manufacturer’s data, the lowest detectable concentration that could be distinguished from the zero standard (LOD) and the limit of quantification (LOQ) were both 1.48 nmol/L at 95% confidence limit. The percentage of cortisone cross reactivity was 3.00%. Intra- and inter-assay variability for cortisol ranged between 1.5% and 4.5% (for concentrations from 2.59 to 48.28 nmol/L) and 5.8% and 7.5% (for concentrations 112.69 and 67.00 nmol/L).

After measurements of salivary cortisol concentrations, three indexes were calculated. The CAR was calculated as the difference between the cortisol level 30 to 45 minutes after waking and the cortisol concentration immediately after awakening, the DCS was estimated as the difference between waking cortisol level and bedtime cortisol level, divided by the time (hours) between these samples and the AUC_G_ was calculated using the trapezoid formula (*9*).

### Statistical analysis

All statistical analysis were performed using SPSS/PASW version 20 (IBM Corp., New York, USA). A simple nonparametric method was used for calculating the reference intervals (RIs) and 90% confidence intervals (90% CIs), following the EP28-A3C recommendations and recently published guidelines in the journal (*8*, *10*).

## Results

Since gender was one of the major determinants of salivary cortisol secretion in the CLASS, it was used as a portioning criterion for dividing the reference sample (*7*, *8*). Table 1 shows the RIs of salivary cortisol measures determined by the nonparametric method in males and females along with 90% CIs of the reference limits. The medians and IQRs are given elsewhere (*7*). The lower limits of RIs determined by the direct method were higher in females (> 10%) than in males for the SCC_0_, SCC_30-45_ and SCC_bedtime_. However, the upper limit of RI for the SCC_bedtime_ was higher (> 10%) in males compared to females. The RIs of the derived salivary cortisol indexes also showed different patterns among males in comparison to females. In the majority of cases, females had higher upper limits of RIs (CAR, DCS) and higher lower limits of RIs (CAR, AUC_G_). The lower limits of RIs for the DCS was higher in males than in females.

**Table 1:** Reference intervals of salivary cortisol measures estimated by the direct method using a non-parametric calculation

**Salivary cortisol**	**Males**				**Females**			
**N**	**Reference interval (95%)**			**N**	**Reference interval (95%)**		
	**LL-CI90**	**LL-UL**	**UL-CI90**		**LL-CI90**	**LL-UL**	**UL-CI90**
SCC_0_, nmol/L	372	3.88 − 5.54	5.28 − 32.73	29.27 − 37.27	596	5.52 − 7.05	5.88 − 36.13^§^	34.07 − 41.07
SCC_30−45_, nmol/L	372	3.90 − 6.57	5.40 − 41.66	36.28 − 45.43	597	7.12 − 8.74	7.97 − 44.93^§^	10.85 − 49.08
SCC_bedtime_, nmol/L	371	1.79 − 2.73	2.30 − 21.93*	18.90 − 25.51	597	2.37 − 2.81	2.56 − 16.94^§^	14.90 − 17.93
CAR, nmol/L	370	- 12.85 – (- 9.49)	- 10.74 − 21.47	18.84 − 24.60	593	- 15.5 – (-10.51)	-13.02 − 28.82^§||^	24.81 − 30.35
DCS, nmol/L per hour	351	-0.70 – (- 0.29)	- 0.40 − 1.60^†^	1.36 − 1.86	568	- 0.36 – (-0.06)	- 0.19 − 1.91^||^	1.77 − 2.15
AUC_G_, nmol/L per hour	349	64.32 − 81.51	71.23 − 471.87	397.58 − 530.74	565	85.81 − 104.65	95.02 − 450.31^§^	425.73 − 481.86
SCC_0_ - cortisol concentrations at awakening. SCC_30−45_ - cortisol concentrations 30 to 45 after awakening. SCC_bedtime_ - cortisol concentrations at bedtime. CAR - cortisol awakening response. DCS - diurnal cortisol slope. AUC_G_ - area under the curve with respect to ground. UL – upper limit. LL – lower limit. CI90 – confidence interval (90%). *UL of males (> 10%) higher than females. ^†^LL of males higher (> 10%) than females. ^||^UL of females (> 10%) higher than males. ^§^LL of females higher (> 10%) than males.								

## Discussion

The data from this study provide RIs for six measures of salivary cortisol for the reference population of late adolescents aged 18 to 21 years. The current study clearly indicates differences between late adolescent males and females in the lower and upper limits of RIs. In the majority of salivary cortisol measures, higher lower limits of RIs were observed among females. This is probably the reason why the average salivary cortisol concentration of this age group is higher in females than males (*5*, *7*).

Reported reference values were calculated for salivary samples collected from January until March. Typically, salivary cortisol values peak in spring and winter (*5*). Therefore, in future studies it would be advisable to establish the circannual reference values (spring and winter *vs.* summer and autumn) for salivary cortisol.

The CLASS reference values for salivary cortisol open the arena for introducing salivary bioscience in the Croatian clinical laboratory practice and provide important data for better understanding of the HPA axis functioning and gender differences in adaptation to stress during late adolescence, both for basic scientists and health practitioners.
